# Adherence to guidelines and protocols in the prehospital and emergency care setting: a systematic review

**DOI:** 10.1186/1757-7241-21-9

**Published:** 2013-02-19

**Authors:** Remco HA Ebben, Lilian CM Vloet, Michael HJ Verhofstad, Sanne Meijer, Joke AJ Mintjes-de Groot, Theo van Achterberg

**Affiliations:** 1 Research group for Acute Care, Faculty of Health and Social Studies, HAN University of Applied Sciences, Verlengde Groenestraat 75, 6525 EJ, Nijmegen, The Netherlands; 2 Canisius Wilhelmina Hospital, Weg door Jonkerbos 100, 6532 SZ, Nijmegen, The Netherlands; 3 Sint Elisabeth Hospital, Hilvarenbeekseweg 60, 5022 GC, Tilburg, The Netherlands; 4 Scientific Institute for Quality of Healthcare, Radboud University Nijmegen Medical Centre, Geert Grooteplein 21, 6525 EZ, Nijmegen, The Netherlands; 5 Research group for Acute Care, Faculty of Health and Social Studies, HAN University of Applied Sciences, PO Box 6960, 6503 GL, Nijmegen, The Netherlands

**Keywords:** Emergency medical technicians [MeSH], Emergency medical services [MeSH], Emergency medicine [MeSH], Emergency nursing [MeSH], Guideline adherence [MeSH]

## Abstract

A gap between guidelines or protocols and clinical practice often exists, which may result in patients not receiving appropriate care. Therefore, the objectives of this systematic review were (1) to give an overview of professionals’ adherence to (inter)national guidelines and protocols in the emergency medical dispatch, prehospital and emergency department (ED) settings, and (2) to explore which factors influencing adherence were described in studies reporting on adherence. PubMed (including MEDLINE), CINAHL, EMBASE and the Cochrane database for systematic reviews were systematically searched. Reference lists of included studies were also searched for eligible studies. Identified articles were screened on title, abstract and year of publication (≥1990) and were included when reporting on adherence in the eligible settings. Following the initial selection, articles were screened full text and included if they concerned adherence to a (inter)national guideline or protocol, and if the time interval between data collection and publication date was <10 years. Finally, articles were assessed on reporting quality. Each step was undertaken by two independent researchers. Thirty-five articles met the criteria, none of these addressed the emergency medical dispatch setting or protocols. Median adherence ranged from 7.8-95% in the prehospital setting, and from 0-98% in the ED setting. In the prehospital setting, recommendations on monitoring came with higher median adherence percentages than treatment recommendations. For both settings, cardiology treatment recommendations came with relatively low median adherence percentages. Eight studies identified patient and organisational factors influencing adherence. The results showed that professionals’ adherence to (inter)national prehospital and emergency department guidelines shows a wide variation, while adherence in the emergency medical dispatch setting is not reported. As insight in influencing factors for adherence in the emergency care settings is minimal, future research should identify such factors to allow the development of strategies to improve adherence and thus improve quality of care.

## Introduction

Clinical practice guidelines and protocols are developed to improve quality of care, to reduce variation of practice and to ensure that evidence is actually used when appropriate [1]. Often, these instruments are developed and disseminated by (inter)national professional organisations [2,3]. A guideline consists of systematically developed recommendations to assist practitioners and patient decisions about appropriate health care for specific clinical circumstances [4]. A guideline recommendation is defined as “any statement that promotes or advocates a particular course of action in clinical care” [5]. To assist implementation of guidelines, a protocol can be developed, which yields a specification of a guideline and exactly formulates how to act and which steps to follow [6]. Despite the existence of guidelines and protocols, a gap between recommended care and clinical practice often exists [7,8]. This is shown in a systematic review on the quality of health care delivered to adults in the United States [9]. Results showed that patients received 54.9% of recommended care, that the proportion of recommended care slightly differed for preventive, acute, and chronic care, and that differences were even larger for different medical functions (screening, diagnosis, treatment and follow-up).

It is suggested that effective implementation should ensure guideline adherence in practice and subsequently lead to improved patient outcomes [5]. Implementation is defined as "a planned process and systematic introduction of innovations or changes of proven value; the aim being that these are given a structural place in professional practice, in the functioning of organisations or in the health care structure" [6]. A systematic review on factors influencing implementation of clinical guidelines concluded that influencing factors were related to the used implementation strategies, and characteristics of the guidelines, professionals, patients and environment [10].

Similar to other settings, guidelines and protocols have become an important aspect of prehospital and emergency care clinical practice [11,12]. Yet, only few studies have investigated to what extent emergency care professionals actually adhere to these instruments [11]. When professionals do not adhere to guidelines and protocols, patients in the prehospital and emergency care settings may not receive appropriate care and quality of care can be threatened.

### Objective

The first objective of this study was to present an overview of professionals' adherence to (inter)national guidelines and protocols in the emergency medical dispatch, prehospital and emergency department (ED) setting. The underlying rationale for choosing these settings is that they are often regarded as 'the chain of emergency care' and that all professionals, irrespective of setting, are expected to provide emergency care as described in guidelines and protocols. The second objective was to explore which factors influencing adherence were described in studies reporting on adherence. This insight can provide valuable input for the development of strategies to successfully implement guidelines and protocols in the emergency care settings.

## Methods

A systematic review of the literature was performed. The review is reported conform the PRISMA statement (Preferred Reporting Items for Systematic Reviews and Meta-Analysis) [13].

### Type of studies

All types of quantitative studies which described adherence to guidelines or protocols in the emergency medical dispatch, prehospital ambulance care and ED settings were included. Studies using self-report methods were excluded as they incorporate a risk of overestimation [14].

### Type of guidelines

Studies describing adherence to (inter)national guidelines and protocols concerning all types of medical conditions in all types of emergency settings in all countries and regions within countries were included. Studies concerning local guidelines and protocols were excluded as it was unclear how they were developed and to what degree they were evidence-based.

### Type of outcome measures

One of the outcome measures of the study had to include adherence quantified as percentage.

### Electronic searches

PubMed (including MEDLINE), CINAHL, EMBASE and the Cochrane database for systematic reviews were searched in June 2010. Search strategies contained ‘terms for professionals’ AND ‘terms for settings’ AND ‘terms for adherence’ AND ‘terms for guidelines/protocols’. Full search strategies per database are given in Appendix 1. Searches were restricted by year of publication (≥1990). No other restrictions were used. In addition to the electronic search, we hand searched reference lists of included articles. We searched the Cochrane database for systematic reviews for both planned and completed reviews on adherence, but found none.

### Selection of studies

All articles were screened on title and abstract by two independent reviewers (RE, LV) and included if the title or abstract described adherence in one of the emergency care settings. After initial selection, remaining articles were screened full text by researchers in two pairs (RE, LV, JM, TvA) and were included if (a) the adherence concerned specified guidelines or protocols, and (b) if the time interval between data collection and publication date of the guideline or protocol did not exceed ten years as non-adherence with outdated recommendations might be justified in these cases. Conference abstracts, editorials, personal communications, or unpublished studies were excluded.

### Quality assessment

To provide a quality indicator, two pairs of independent researchers assessed reporting quality of all included studies (RE, LV, JM, TvA). For this assessment we developed a checklist, which was based on the STROBE statement to assess the reporting of cohort and cross sectional studies [15] and the TREND statement to assess the reporting of interventional studies [16]. The checklist consisted of ten items to assess quality: (1) objective, (2) key elements, (3) setting, locations and dates, (4) eligibility criteria, (5) outcomes, (6) data sources and methods, (7) data analysis and statistical methods, (8) number of participants, (9) characteristics of participants, and (10) main results. For each item an article could score a 'described' (1 point), 'partly described' (0.5 point), or 'not described' (0 points). All included articles were rated on a scale from 1 (poor study report) to maximum 10 (excellent study report).

### Data extraction

From each article (a) the number of guideline or protocol recommendations described, and (b) adherence percentages for each recommendation were extracted. In case of multiple measurements regarding one recommendation, multiple adherence percentages were extracted. In case of a pre-test post-test design for the evaluation of quality improvement, only the pre-test percentages were extracted as we focused on actual care rather than effects of quality improvement strategies. From each study, the guideline and protocol recommendations were categorised into medical condition (cardiology, pulmonology, neurology, infectious diseases, or other) and into type of medical function (diagnostic, treatment, monitoring, or organisational) (Table 1). Categorisation was done as 'medical condition' and 'medical function' have been indicated as influencing factors for guideline adherence previously [10,17]. The median adherence for each recommendation was extracted or calculated. Additionally, factors influencing adherence were extracted when a statistically significant relationship between the factor and adherence was demonstrated in the article. Non-significant factors are not shown. The corresponding author of one study was contacted through e-mail to clarify and confirm results.

**Table 1:** Categories of guideline recommendations classified by medical function

**Medical function**	**Examples**
Diagnostic	1. Evaluate arterial blood gas for patients with acute exacerbations of COPD [19]
	2. Obtain blood culture in case of a child with fever [42]
Treatment	1. Administer benzyl penicillin if a patient has a non-blanching purpuric rash [25]
	2. Administer epinephrine 1 mg intravenous, intraosseous or endotracheal if a patient has cardiac arrest [27]
Monitoring	1. Monitor blood pressure and SaO_2_ at least once for a patient with cardiac arrest [26]
	2. Monitor EtCO_2_ for a patient with cardiac arrest [26]
Organisational (referral, documentation)	1. Refer to an allergist in case of a severe allergic reaction [49]
	2. Document asthma severity (mild, moderate, severe) [35]

All data were extracted by two independent researchers (RE, SM). To assess inter-rater reliability, the overall agreement percentages were calculated on number of guideline or protocol recommendations and adherence percentages. For articles concerning the prehospital care setting, these were 93% and 83% respectively, and for articles concerning the ED setting these were 90% and 85%. Since the heterogeneity of study designs, guideline recommendations, medical conditions, and medical functions was substantial, a meta-analysis was not feasible. Instead, we extensively analysed the studies and conducted a qualitative synthesis.

## Results

### Description of the studies

**Figure 1:**
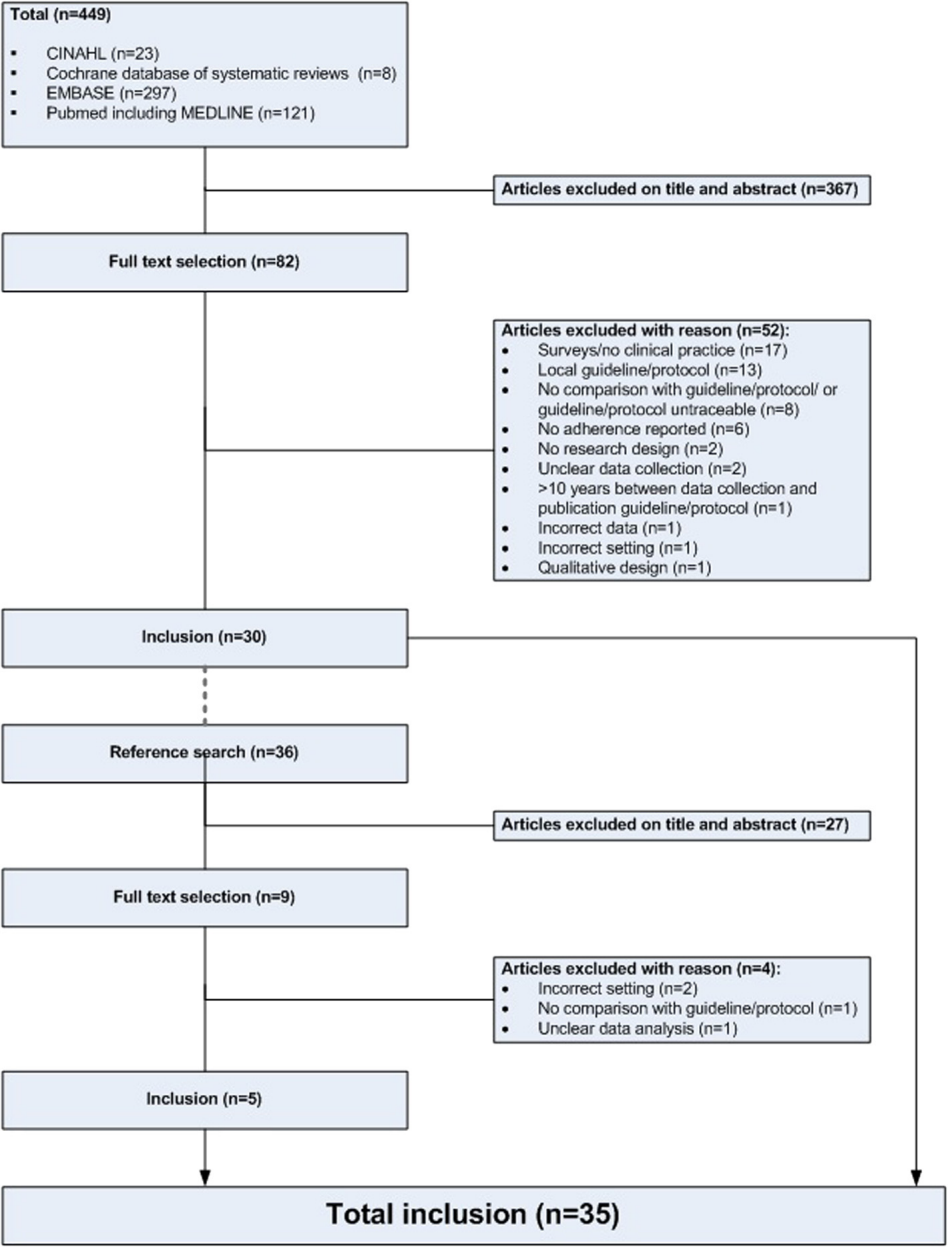
Inclusion of studies.

The electronic search identified 30 articles meeting the inclusion criteria. In addition, another five articles were included by searching the reference lists (Figure 1). Of the included articles (n=35), 24 used retrospective, 9 used prospective, and 2 used cross sectional methods. Eighteen studies were multicentric and seventeen were monocentric, with 31 covering adults and 4 covering children. The studies were conducted in North America (n=19), Europe (n=13), Australia (n=2), and Asia (n=1). One study described adherence in the prehospital setting as well as in the ED setting [18] and results of this study are therefore presented in both the prehospital and ED result sections. All studies described adherence to (inter)national guidelines No studies on adherence to (inter)national protocols were identified. Seven studies assessed adherence to a guideline which was not developed in their own country [18-24]. The quality assessment revealed 34 articles of excellent or good reporting quality (excellent report - ten points, very good report - nine points, good report - eight points). Only one article was of moderate reporting quality with seven points [25]. As only the reporting quality was assessed, no articles were excluded on the basis of this quality assessment. Further details of the included studies are described in Table 2.

**Table 2:** Characteristics of included studies (n=35)

**First author**	**Design**	**Methods**	**Monocenter/multicenter**	**Professionals**	**Patients**	**Guideline (year of publication)**	**Quality**
**(Year)**							
**Country**							
**Prehospital**							
Caulfield	Retrospective, descriptive	Prehospital record review	Monocenter: 1 EMS	HEMS paramedics	100 patients with traumatic brain injury	Brain Trauma Foundation Guideline for prehospital management of patients with traumatic brain injury (2007)	9.5
(2009)							
USA							
Cooke	Retrospective, descriptive	Patient report forms	Multicenter: 19 EMSs	Paramedics	69 patients with suspected meningococcal septicemia	Joint Royal Colleges Ambulance Liaison Committee Clinical Guidelines for the administration of benzyl penicillin for suspected diagnosis of meningococcal septicemia (2003)	7
(2005)							
UK							
Franschman 2009 The Netherlands	Retrospective, descriptive	Medical record review	Monocenter: 1 EMS	Ambulance nurses EMS physicians	127 patients with traumatic brain injury	Brain Trauma Foundation Guideline for prehospital management of patients with traumatic brain injury (2007)	9
						Dutch Ambulance Care National Protocol (2007)	

Hale	Retrospective, descriptive	Prehospital record review	Monocenter: 1 EMS	Not specified	1022 patients who received O_2_	Joint Royal Colleges Ambulance Liaison Committee Clinical Guidelines for the administration of oxygen (2007)	8.5
(2008)							
UK							
Jeremie	Prospective, descriptive	Prehospital record review	Multicenter: 3 EMSs	Anesthesiologists Emergency physicians	143 patients who were sedated and intubated	SFAR Recommendations for sedation: analgesia in out-of-emergency medicine (2000)	10
(2005)							
France							
Kirves	Retrospective, cohort	Prehospital record review	Multicenter: >75 EMSs	Paramedics EMS physicians	157 patients with cardiac arrest	The Subdivision of Emergency Medicine of Finnish Society of Anaesthesiologists, Finnish Resuscitation Council and Red Cross of Finland. Resuscitation guidelines (2002)	9
(2007)							
Finland							
Scliopou	Retrospective, descriptive	Database review	Multicenter: 35 EMSs	Paramedics	70 patients with cardiac arrest	American Heart Association Advanced cardiac Life Support Guidelines (2000)	10
(2005)							
USA							
Thomas	Prospective, descriptive	Data collection chart	Monocenter: 1 EMS	HEMS nurses HEMS paramedics	37 patients with traumatic brain injury	Brain Trauma Foundation guidelines for the Management of Severe Head Injury (1995)	10
(2002)							
USA							
Wik (2005) Norway/Sweden/UK	Prospective, case series	Data cards	Multicenter: 3 EMSs	Nurse anesthesists Paramedics	176 patients with cardiac arrest	Guidelines for Cardiopulmonary Resuscitation and Emergency Cardiovascular Care: International Consensus on Science (2000)	10
						International guidelines for CPR and ECCL: a consensus on science (2000)	
**Prehospital & Emergency Department**							
							
Charpentier	Prospective, cohort	Case report form	Multicenter: 1 UH, 8 EMSs, 26 MICUs, 37 EDs, 22 CICUs	Emergency physicians	1277 patients with ST-segment elevation myocardial infarction	American College of Cardiology/American Heart Association guidelines for the management of patients with acute myocardial infarction (1999)	10
(2009)							
France							
**Emergency Department**							
Atreja	Retrospective, descriptive	Chart review	Monocenter: 1 ED	Emergency physicians	94 patients with an elevated international normalized ratio (INR)	American College of Chest Physicians recommendations for antithrombotic therapy for prevention and treatment of thrombosis (2001)	10
(2005)							
USA							
Clark	Retrospective, cohort	Medical record review	Multicenter: 21 EDs	Not specified	678 patients with allergic reaction to food	American academy of allergy, asthma, & immunology guideline for the management of food allergy (2003)	10
(2004)							
USA & Canada							
Cydulka (2003) USA/Canada	Prospective, cohort	Medical record review Telephone interviews	Multicenter: 29 EDs	Not specified	397 patients with exacerbation COPD	American thoracic society standards for the diagnosis and care of patients with chronic obstructive pulmonary disease (COPD) and asthma (1987)	10
						British Thoracic Society guidelines for the management of chronic obstructive pulmonary	

De Miguel-Yanes	Retrospective, cohort	Medical record review	Monocenter: 1 ED	Not specified	53 patients with suspected sepsis	Surviving sepsis campaign guidelines for management of severe sepsis and septic shock (2004)	9.5
(2006)							
Spain							
Doherty	Retrospective, pre-test post-test	Database review	Multicenter: 2 EDs	Not specified	215 patients with asthma	NSW Department of Health guideline for the optimal treatment of chronic respiratory diseases (2003)	10
(2007)							
Australia							
Elkharrat	Prospective, pre-test post test	Data collection chart	Monocenter: 1 ED	Not specified	389 patients with open wounds	World Health Organisation guideline for antitetanus prophylaxis (1992)	10
(1999)							
France							
Ferguson	Retrospective, cohort	Medical record review	Monocenter: 1 ED	Pediatric emergency physicians	167 children with fever	Agency for Health Care Policy and Research guideline for the management of infants and children 0 to 36 months of age with fever without source (1993)	9.5
(2012)							
USA							
Grant	Retrospective, descriptive	Medical record review	Monocenter: 1 ED	Not specified	473 patients with acute pain	British Association of Accident and Emergency Medicine guideline for the management of pain in adults (2005)	10
(2006)							
UK							
Jain (2002) USA	Retrospective, descriptive	Medical record review	Monocenter: 1 ED	Pediatric residents	229 children with fever	Agency for Health Care Policy and Research guideline for the management of infants and children 0 to 36 months of age with fever without source (1993)	9.5
				Fellows			
				Nurse practitioners			
Kelly	Prospective, descriptive	Data collection chart	Multicenter: 38 EDs	Not specified	1340 patients with acute asthma	National Asthma Campaign asthma management guideline (1998)	9.5
(2013)							
Australia							
Lee (2001) Taiwan	Retrospective, cohort	Medical record review	Multicenter: 6 EDs	Emergency physicians	120 patients with acute asthma	1. British Thotacic Society guidelines I & II for the management of asthma in adults (1990&1993)	9
						2. National Heart, Lung and Blood Institute guideline for the diagnosis and management of asthma (1991 & 1994 & 1997)	
						3. Asthma management guidelines and therapeutic Issues relating to the treatment of asthma. Chest (1999)	
Mansbach (2007) USA	Prospective, cohort	Medical record review	Multicenter: 17 EDs	Not specified	624 children with bronchiolitis	American Academy of Pediatrics Committee on Infectious Diseases and Committee of Fetus and Newborn guidelines for prevention of respiratory syncytial virus infections: indications for the use of palivizumab and update on the use of RSV-IGIV (1998)	9
		Interviews					

Milks	Retrospective, descriptive	Medical record review	Monocenter: 1 ED	Not specified	181 patients with asthma	National Heart, Lung and Blood Institute guideline for the diagnosis and management of asthma (1991)	8
(1999)							
USA							
Muayqil	Retrospective, descriptive	Medical record review	Monocenter: 1 ED	Emergency physicians	45 patients with convulsive status epilepticus	Epilepsy Foundation of America guidelines for the management convulsive status epilepticus (1993)	10
(2007)							
Canada							
Musacchio	Retrospective, descriptive	Medical record review	Monocenter: 1 ED	Not specified	163 patients with urinary tract infections, urinary symptoms or sexually transmitted disease	Center for Disease Control and Prevention: guideline for treatment of sexually transmitted diseases (2006)	9
(2009)							
USA							
Pham (2007) USA	Cross sectional, descriptive	Database review	Multicenter: 544 EDs	Not specified	1492 patients with acute myocardial infarction	Center for Medicare and Medicaid Services. Specification manual for national hospital quality measures for acute myocardial infarction and asthma (2007)	10
					3955 patients with pneumonia		

Reid (2000) Canada	Retrospective, descriptive	Medical record review	Multicenter: 3 EDs	Emergency physicians	130 patients with asthma	National guideline for the emergency management of asthma in adults (1996)	10
					Emergency nurses		

Roy (2006) France & Belgium	Prospective, cohort	Data collection chart	Multicenter: 117 EDs	Emergency physicians	1529 patients with suspected pulmonary embolism	American College of Emergency Physicians Clinical Policies Committee. Clinical policy: critical issues in the evaluation and management of adult patients presenting with suspected pulmonary embolism (2003)	9.5
						British Thoracic Society guidelines for the management of suspected acute pulmonary embolism (2003)	
						European Society of Cardiology Guidelines on diagnosis and management of acute pulmonary embolism (2000)	
Salmeron (2001) France	Prospective, cohort	Data collection chart	Multicenter: 37 EDs	Emergency physicians	4087 patients with acute asthma	1. National Asthma Education and Prevention Program guidelines for the diagnosis and the management of asthma (1997)	10
						2. British guidelines on asthma management, 1995 review and position statement (1997)	

Shaked	Retrospective, descriptive	Medical record review	Monocenter: 1 E	Not specified	56 children with febrile seizure	American Academy of Pediatrics (AAP) Practice Parameter: the neurodiagnostic evaluation of the child with a first simple febrile seizure (1996)	10
(2009)							
USA							
Teismann	Retrospective, descriptive	Medical record review	Monocenter: 1 ED	Emergency residents Physician assistants	553 patients with suspected venous thromboembolism	American College of Emergency Physicians Clinical Policies Subcommittee on Suspected Pulmonary Embolism, evaluation and management of adult patients presenting with suspected pulmonary embolism (2003)	9
(2009)							
USA							
Thakore	Retrospective, descriptive	Medical record review	Monocenter: 1 ED	Not specified	100 patients with syncope	American college of physicians guideline for management of patients with syncope (1997)	9
(1999)							
Scotland							
Trzeciak	Retrospective, cohort	Medical record review	Monocenter: 1 ED	Emergency physicians	22 patients with confirmed or suspected sepsis	Surviving sepsis campaign guidelines for management of severe sepsis and septic shock (2004)	10
(2006)							
USA							
Tsai (2009) USA	Retrospective, cohort	Medical record review	Multicenter: 2 EDs	Emergency physicians	272 patients with COPD	Global Initiative for Chronic Obstructive Lung Disease guidelines for the diagnosis, management, and prevention of chronic obstructive pulmonary disease (2001)	10
		Interview				American College of Physicians guidelines for Management of acute exacerbations of chronic obstructive pulmonary disease (2001)	
						American Thoracic Society and European Respiratory Society joint guidelines Standards for the diagnosis and treatment of patients with COPD (2004)	
Wright	Retrospective, descriptive	Medical record review	Monocenter: 1 ED	Emergency physicians	244 patients who received vancomycin	Center for Disease Control and Prevention: Recommendations for preventing the spread of vancomycin resistance: Recommendations of the Hospital Infection Control Practices Advisory Committee (1995)	10
(1998)							
USA							

Abbreviations: *CICU* Cardiac Intensive Care Unit, *ED* Emergency Department, *EMS* Emergency Medical Service, *HEMS* Helicopter Emergency Medical Service, *MICU* Mobile Intensive Care Unit, *UH* University Hospital.

Quality: assessed on a scale from 0 (poor quality) to 10 (excellent quality).

### Emergency medical dispatch

Our electronic search strategy and reference search did not identify any eligible studies in the emergency medical dispatch setting.

### Prehospital setting

Ten studies were identified describing adherence to (inter)national guidelines in the prehospital setting. These guidelines covered cardiology [18,26-28], pulmonology [29], neurology [30-33], and infectious diseases [25] (Table 3). Professionals included emergency physicians, anesthesiologists, ambulance nurses, nurse anesthesists, emergency medical technicians (EMT), and helicopter emergency medical service (HEMS) paramedics. Four studies were monocentric and six were multicentric. Seven studies were conducted in Europe and the remaining three in North America.

**Table 3:** Guideline topics

**Medical condition**	**Prehospital setting**	**Emergency department setting**
*Cardiology*	Cardiac arrest [26-28]	Myocardial infarction [18,34]
	Myocardial infarction [18]	
*Neurology*	Sedation [32]	Convulsive status epilepticus [21]
	Traumatic brain injury [30-33]	Syncope [24]
*Pulmonology*	Oxygen administration [29]	Bronchiolitis [37]
		Asthma [20,23,35,36,38,39]
		COPD [19,40]
		Pneumonia [34]
*Infectious diseases*	Meningococcal septicaemia [25]	Antibiotic therapy [47]
		Antitetanus prophylaxis [43]
		Fever [42]
		Febrile seizures [44,45]
		Sepsis [41,46]
*Other*	*-*	Allergic reactions to food [49]
		Antithrombotic therapy [48]
		Pain [50]
		Pulmonary and venous embolisms [22,52]
		Urinary complaints/sexually transmitted diseases [51]

From the ten articles, a total of 40 recommendations were extracted. Four (10%) were monitoring recommendations and 36 (90%) were treatment recommendations. On these 40 recommendations, a total of 12 median adherence percentages were extracted or calculated, of which 2 (17%) were monitoring percentages, and 10 (83%) were treatment percentages. The distribution of the percentages across the different medical conditions and types of recommendations is displayed in Additional file 1: Figure 2.

**Figure 2:**
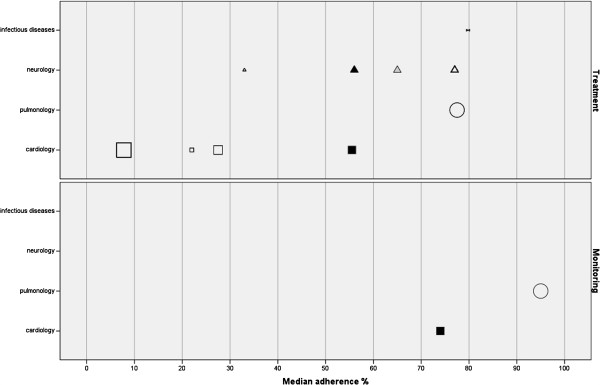
Adherence prehospital setting.

Figure 2 shows median adherence percentages in the prehospital setting varying from 7.8% to 95%. The three lowest median adherence percentages (7.8%, 22%, 27.5%) came with cardiology treatment recommendations related to myocardial infarction [18] and cardiac arrest [27,28], whereas the three highest median adherence percentages (77.5%, 79.8%, 95%) came with treatment recommendations related to oxygen administration [29] and septicaemia [25], and to one monitoring recommendation related to oxygen administration [29]. Looking at medical functions, monitoring recommendations came with less variation in adherence when compared to the treatment recommendations, and monitoring recommendations came with higher median adherence percentages. Regarding the medical conditions, cardiology treatment recommendations are less often adhered to than treatment recommendations for other medical conditions.

### Emergency department setting

Twenty-six studies describing adherence to (inter)national guidelines in the ED setting were identified. These guidelines covered cardiology [18,34], pulmonology [19,20,23,34-40], neurology [21,24], infectious diseases [41-47], and 'other' conditions [22,48-52] (Table 3). Professionals were (paediatric) emergency physicians, medical fellows, emergency nurses, and nurse practitioners. Fourteen studies were monocentric and twelve were multicentric. Sixteen studies were conducted in North America, seven in Europe, two in Australia, and one in Asia.

From the 26 studies, a total of 161 recommendations were extracted. Fifty-one (32%) were diagnostic recommendations, one (<1%) was a monitoring recommendation, 102 (63%) were treatment recommendations, and seven (4%) were organisational recommendations. On these 161 recommendations, a total of 40 median adherence percentages were extracted or calculated. Fourteen (35%) were percentages on the uptake of recommendations for diagnostics, one (2.5%) was a percentage for adherence to a recommendation on monitoring, 20 (50%) were percentages for the uptake of treatment recommendations, and five (12.5%) were adherence percentages for organisational recommendations. The distribution of the percentages across the different medical conditions and types of recommendations is displayed in Additional file 2: Figure 3.

**Figure 3:**
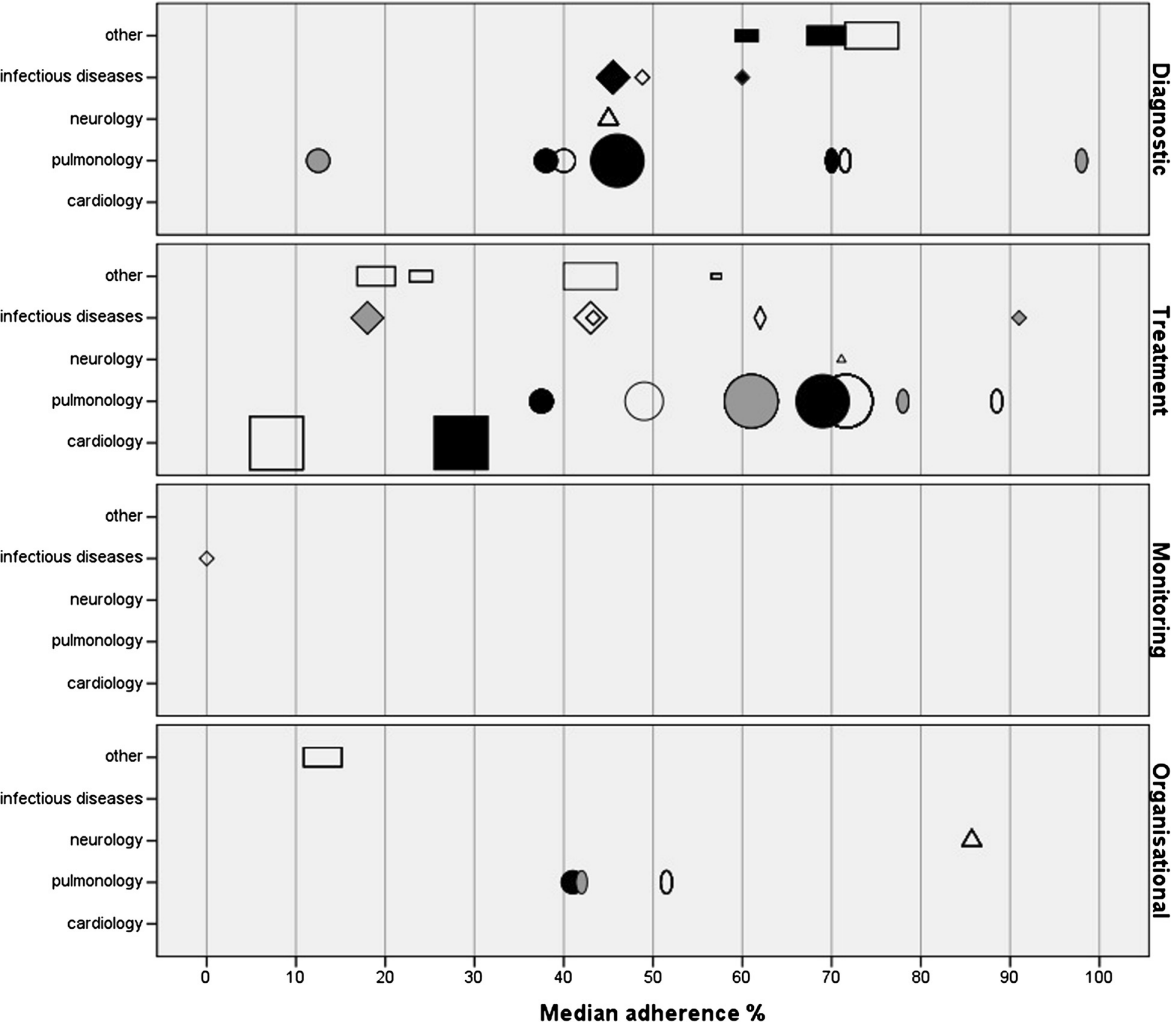
Adherence ED setting.

Figure 3 shows a wide variation in adherence percentages in the ED setting, varying from 0% to 98%. The three lowest median adherence percentages (0%, 7.8%, 12.5%) came with a monitoring recommendation related to sepsis [41], a treatment recommendation related to myocardial infarction [18], and a diagnostic recommendation related to asthma [20]. The highest median adherence percentages (88.5%, 91%, 98%) came with a diagnostic recommendation related to COPD [40], and treatment recommendations related to asthma [38] and sepsis [46]. Looking at medical functions, diagnostic and organisational recommendations came with higher median adherence percentages compared to the treatment recommendations. Among medical conditions, pulmonary treatment recommendations came with higher median adherence percentages, and cardiology treatment recommendations came with lower median adherence percentages compared to other conditions.

### Influencing factors

Eight studies reported factors influencing adherence [18,20,22,26,34,37,42,51]. These factors were related to the patient (age, race, sex, weight, time of presentation, insurance status, current disease/condition and comorbidity) and to the organisation (presence of an emergency physician, ownership (non-federal or governmental) hospital/ED and location) (Table 4). When categorised along medical conditions, the patient related influencing factors had different directions and no clear patterns existed, although male sex, lower age and a disease specific condition (rhythm on the electrocardiogram) seemed to positively influence adherence to cardiology guidelines. As for organisational factors, there seemed to be a pattern that treatment in a governmental or non-federal ED negatively influences adherence to (inter)national guidelines.

**Table 4:** Influencing factors

**Domain**	**Influencing factor**	**Medical condition**
Patient characteristics	Age	*Cardiology*
		• Patients with ST-segment elevation myocardial infarction aged ≤75 years were more likely to receive care in accordance with the guideline [18]
		• Patients with acute myocardial infarction aged <55 years were more likely to receive aspirin [34]
		*Pulmonology*
		• Patients with pneumonia aged <18 years were more likely to receive recommended antibiotics [34]
		• Patients with pneumonia aged <18 years were less likely to be monitored with pulse oximetry [34]
		• Patients with suspected pulmonary embolism aged >75 years were less likely to be diagnosed in accordance with the guideline [22]
		• Children with bronchiolitis whose gestational age was 30 weeks were more likely to receive palivizumab compared to children whose gestational age was 32 weeks [37]
		*Other*
		• Patients with urinary complaints aged >19 years were more likely to be taken their sexual history [51]
		• Children with fever who were aged 28–59 days were more likely to receive complete blood cell count, blood culture, urine culture, cerebrospinal fluid culture and viral studies compared to children who were aged 60–90 days [42]
	Gender	*Cardiology*
		• Male patients with acute myocardial infarction were more likely to receive ß-blockers [34]
		• Male patients with cardiac arrest were more likely to receive treatment in accordance with the guidelines [26]
	Weight	*Pulmonology*
		• Children with bronchiolitis with birth-weight <3lbs were more likely to receive palivizumab [37]
	Current disease/condition	*Cardiology*
		• Patients with ST-segment elevation myocardial infarction with a symptom onset 08.00-20.00 were more likely to receive care in accordance with the guideline than patients with a symptom onset 20.00-08.00 [18]
		• Patients with ST-segment elevation myocardial infarction with a typical STEMI on the ECG were more likely to receive care in accordance with the guideline compared to patients without a typical STEMI on the ECG [18]
		• Patients with cardiac arrest of whom the arrest was witnessed or with an initial rhythm of VF/VT were more likely to receive care in accordance with the guideline than patients with an unwitnessed arrest of initial rhythm other than VF/VT [26]
		• Patients with cardiac arrest with a longer time interval between return of spontaneous circulation and hospital admission were more likely to receive care in accordance with the guideline compared to patients with a shorter time interval [26]
		*Pulmonology*
		• Patients with suspected pulmonary embolism currently receiving anticoagulation were less likely to be diagnosed in accordance with the guideline compared to patients with anticoagulation [22]
		• Children with bronchiolitis with a history of wheezing were more likely to receive palivizumab than patients without a history of wheezing [37]
		*Other*
		• Patients with urinary complaints with a history of fever were more likely to be taken their sexual history than patients without a history of fever [51]
		• Patients with urinary complaints with genital discharge were more likely to be taken their sexual history than patients without genital discharge [51]
	Race	*Cardiology*
		• Patients with acute myocardial infarction of Hispanic race were less likely to receive aspirin compared to patients of white or nonwhite race [34]
		*Pulmonology*
		• Patients with pneumonia of nonwhite race were less likely to be monitored with pulse oximetry compared to patients of white or hispanic race [34]
	Insurance	*Cardiology*
		• Patients with acute myocardial infarction with a private insurance were more likely to receive aspirin than patients with a medicare or Medicaid insurance [34]
		*Pulmonology*
		• Patients with pneumonia with a private insurance were more likely to receive antibiotics than patients with a medicare of Medicaid insurance [34]
	Comorbidity	*Cardiology*
		• Patients with cardiac arrest with a prior neurological disease were less likely to receive care in accordance with the guideline compared to patients without prior neurological disease [26]
		*Pulmonology*
		• Patients with suspected pulmonary embolism with known heart failure, known chronic lung disease or current/recent pregnancy were less likely to be diagnosed in accordance with the guideline than patients without known heart failure, chronic lung disease or current/recent pregnancy [22]
		• Patients with suspected pulmonary embolism with previous thromboembolism were more likely to be diagnosed in accordance with the guideline than patients without previous thromboembolism [22]
	Time of presentation	*Other*
		• Patients with urinary complaints who presented in the evening were more likely to be taken their sexual history compared to patients who presented in over daytime [51]
Organisational factors	Location	*Cardiology*
		• Patients with ST-segment elevation myocardial infarction treated in an urban ED were more likely to be treated in accordance with the guideline compared to patients treated in a rural ED [18]
		• Patients with acute myocardial infarction treated in a Midwest or Southern ED were less likely to receive ß-blockers compared to patients treated in a northeast or west ED [34]
		*Pulmonology*
		• Patients with pneumonia treated in a Southern ED are less likely to receive antibiotics compared to patient treated in a northeast, west or midwest ED [34]
		• Patients with pneumonia treated in a metropolitan ED are more likely to receive antibiotics and are more likely to be monitored with pulse oximetry compared to patients in a non-metropolitan ED [34]
		• Patients with asthma treated in medical centres were more likely to be diagnosed with oximetry or arterial blood gas compared to patients in regional and district EDs [20]
	Presence of a physician	*Cardiology*
		• Patients with cardiac arrest where a prehospital physician was present on scene were more likely to receive care in accordance with the guideline than patients without prehospital physician presence [26]
	Ownership of the ED	*Cardiology*
		• Patients with acute myocardial infarction treated in an ED with governmental or non-federal ownership are less likely to receive aspirin than patients treated in an nonprofit or proprietary ED [34]
		*Pulmonology*
		• Patients with pneumonia treated in an ED with governmental or non-federal ownership are less likely to receive antibiotics compared to patients treated in an nonprofit or proprietary ED [34]

## Discussion

This systematic review aimed to give an overview of professionals' adherence to (inter)national guidelines and protocols in the emergency medical dispatch, prehospital ambulance and ED settings. In addition, factors influencing adherence were explored. Thirty-five articles describing adherence to (inter)national prehospital and ED guidelines were identified. No studies describing adherence to protocols or studies in the emergency medical dispatch setting were identified. Despite the life-threatening and urgent conditions covered by the guidelines, results showed a wide variation in adherence. Extracted factors influencing adherence were related to the patient and to the organisation.

For both the prehospital and ED setting adherence showed a wide variation. Suboptimal adherence has also been shown in other critical care fields, such as the intensive care unit [53,54] and the recovery room [55,56], but also on more general topics as hand hygiene [57] and medication safety [58]. It is possible that the wide variation in adherence is due to often poor evidence-based prehospital guidelines [59], to differences in guideline quality or due to justified deviations as guidelines have to be tailored to unique patients. Unjustified deviations may also contribute to this wide variation in adherence, as situations where guideline deviations are desired are unclear [60]. Specifically regarding the ED setting, another reason for suboptimal adherence may be that guidance for some ED presentations are derived from guidelines of specialties outside the ED as ED guidelines are lacking. As guideline development programmes increasingly become evidence based [61] and guidelines represent the standard of care, our results probably also imply that many patients in the prehospital and ED setting do not receive appropriate care.

Guideline recommendations were extracted to categorise the adherence percentages into recommendation categories in relation to medical function and medical condition. For medical function in the prehospital setting, monitoring recommendations came with higher adherence percentages compared to treatment recommendations. In the ED setting, diagnostic and organisational recommendations came with higher median adherence percentages compared to treatment recommendations. This may indicate that the type of medical function influences adherence to (inter) national guideline recommendations. This result is supported by a previous non-emergency care review, which showed that characteristics of the guideline recommendations (medical condition, type of procedure, complexity) influence guideline adherence [17]. A possible explanation for the large variation in adherence rates for different types of guideline recommendations may be the existence of barriers specifically for individual recommendations rather than guidelines as a whole [5]. For instance, the strength of evidence and the impact on patient outcomes may vary across individual recommendations. Another explanation may be that guidelines contain too many recommendations to adhere to, or that EMSs and EDs are not able to implement all recommendations at the same time and make choices. If this is the case, guidelines could be translated into more efficient, practical and feasible protocols, algorithms, and decision trees.

In addition to differences for types of medical functions of guideline recommendations, variation in adherence percentages for medical conditions was observed. This variation has been reported previously [17]. Especially the cardiology and 'other' guidelines came with lower adherence percentages compared to other medical conditions. These cardiology guidelines cover cardiac arrest and ST-elevation myocardial infarction, two conditions known for their high mortality rates [62,63], while pain ('other' guideline) is reported to be the main complaint for patients to use emergency care [64].

Factors influencing adherence were reported in eight studies [18,20,22,26,34,37,42,51]. These factors can be clustered into factors related to the patient and to the organisation. No professional related factors were studied, which is remarkable as previous studies showed that individual experience, professional autonomy, attitudes and believes also determine to what degree professionals adhere to a guideline and that additional, individual training for ambulance nurses improves adherence to national prehospital protocols [65-67]. Additional research is needed, focussing on the perspectives of professionals, patients, organisations, social environment and characteristics of guidelines and protocols [7]. This knowledge can be used to develop and revise guidelines and protocols [68] and to tailor strategies to improve adherence. It is even argued that these strategies should be tailored to individual guideline recommendations instead of the guideline as a whole [5]. A systematic review showed that strategies tailored to identified barriers are effective to improve professional practice [69]. For the emergency care setting, previous studies showed that strategies tailored to influencing factors improve adherence to guidelines and protocols for patients with asthma, acute coronary syndromes and ST-elevation myocardial infarction [35,70,71]. To monitor adherence and assess effectiveness of implementation strategies it is recommended that guidelines contain clinical indicators [72]. These indicators have shown to be useful to assess and monitor guideline adherence [73]. Therefore, quality indicators should be part of the guideline development process or should be integrated in existing guidelines.

Besides implementations strategies, solid evidence based recommendations and a clear relationship between guideline adherence and patient outcomes may be the strongest motivators for emergency care professionals to adhere to guidelines. Generally, it is stated that especially prehospital care lacks strong evidence and clear indicators to measure effectiveness [74]. In this review, four studies assessed the relationship between adherence and patient outcomes. Three of these showed that adherence to guidelines improves patient outcomes by decreasing mortality and adverse events for patients with ST-segment elevation myocardial infarction, cardiac arrest and suspected pulmonary embolism [18,22,26]. However, the limited number of studies assessing the relationship prevents us from drawing firm conclusions. Therefore, future research should focus on the relationship between guideline adherence and patient outcomes.

We did not find studies in the emergency medical dispatch setting which met our inclusion criteria. Since the dispatch center is the first in the 'chain of emergency care', adherence to dispatch guidelines and protocols is important to correctly identify and prioritize the most urgent patients. Therefore, we recommend additional research on guideline and protocol adherence in this specific setting. One article assessed adherence in two consecutive emergency settings [18]. It is widely recognized that patients enter a 'chain of emergency care', and therefore assessment of adherence to guidelines and protocols in consecutive settings seems reasonable.

### Limitations of included studies

The included studies predominantly had a retrospective design and used patient records or databases to retrieve their data. These methods incorporate a high risk of bias. The second problem we faced was the fact that the included studies incorporated a variability of guidelines, medical conditions, medical functions, designs, and methods, and that some studies assessed adherence to ‘foreign’ guidelines. Therefore, an overall comparison between the studies was difficult. Third, the included studies used several synonyms and definitions of adherence, including compliance, deviation, and ‘guideline follow-up’. Literature shows no clear and widely used definition of adherence, while agreement on a useful definition would assist research. Finally, none of the included studies addressed the seriousness of the deviations, which may have been useful as previous research indicated that 45% of guideline deviations can be categorised as serious or very serious [68].

### Study limitations

A limitation regards the assessment of reporting quality of the included articles, for which we used a checklist based on the STROBE and TREND statements. We are aware that the intended goal of these statements is to provide guidance on reporting research rather than assessing study quality, but adequate quality assessment tools for observational studies are lacking [75]. Furthermore, the differences in settings, personnel, disease processes, and guidelines made interpretation of the results exceedingly challenging.

## Conclusion

Despite the often life-threatening and vital topics of the guidelines, adherence to (inter)national prehospital and ED guidelines showed a wide variation and ranges from 7.8-95% and 0-98% respectively. Research on adherence in the emergency medical dispatch setting is lacking. In the prehospital setting monitoring recommendations came with higher adherence percentages than treatment recommendations. For both settings, the cardiology treatment recommendations were less adhered to than recommendations for other medical conditions. These results indicate that the medical function and medical condition into which a guideline recommendation can be categorised might influence adherence. Further factors influencing adherence were related to the patient and the organisation. Factors related to professionals were not found. Further research should focus on identifying factors influencing adherence, taking into account the perspectives of the professional, patients, organisation, and characteristics of the guidelines. On the basis of these influencing factors, strategies can be developed to improve adherence to prehospital and ED guidelines, with the ultimate goal to ensure that patients receive appropriate care.

## Competing interests

The authors declare that they have no competing interests.

## Authors’ contributions

Study design (RE, LV, MV, JM, TvA). Data collection and analysis (RE, LV, MV, SM, JM, TvA). Quality assessment (RE, LV, JM, TvA). Manuscript preparation (RE, LV, SM, MV, JM, TvA). All authors read and approved the final manuscript.

## Supplementary Material

Additional file 1 Legend Figure 2 prehospital setting. Click here for file

Additional file 2 Legend Figure 3 ED setting. Click here for file
