# Supplementary material for: Adherence to guidelines and protocols in the prehospital and emergency care setting: a systematic review
Source: Scand J Trauma Resusc Emerg Med. 2013 Feb 19;21:9. doi: 10.1186/1757-7241-21-9 (PMC3599067; doi:10.1186/1757-7241-21-9)
Supplement: Additional file 1 — Legend Figure 2 prehospital setting. [file 1757-7241-21-9-S1.doc]

| **Legend Figure 2 prehospital setting** | | | |
| --- | --- | --- | --- |
| **Medical condition** | **> 200 patients** | **100-200 patients** | **<100 patients** |
| **Cardiology** | [18] | [28]  [26] | [27] |
| **Pulmonology** | [29] |  |  |
| **Neurology** |  | [32]  [31]  [30] | [33] |
| **Infectious diseases** |  |  | [25] |
| Study [18] is also displayed in Figure 3 | | | |
