# Supplementary material for: Adherence to guidelines and protocols in the prehospital and emergency care setting: a systematic review
Source: Scand J Trauma Resusc Emerg Med. 2013 Feb 19;21:9. doi: 10.1186/1757-7241-21-9 (PMC3599067; doi:10.1186/1757-7241-21-9)
Supplement: Additional file 2 — Legend Figure 3 ED setting. [file 1757-7241-21-9-S2.doc]

| **Legend Figure 3 ED setting** | | | | |
| --- | --- | --- | --- | --- |
| **Medical condition** | **> 1000 patients** | **500-1000 patients** | **100-499 patients** | **<100 patients** |
| **Cardiology** | [18]  [34] |  |  |  |
| **Pulmonology** | [36]  [34]  [23] | [37] | [19]  [35]  [20]  [38]  [39]  [40] |  |
| **Neurology** |  |  | [24] | [21] |
| **Infectious diseases** |  |  | [43]  [42]  [44]  [47] | [41]  [45]  [46] |
| **Other** | [22] | [49]  [52] | [50]  [51] | [48] |
| Study [18] is also displayed in Figure 2 | | | | |
